# Flexible 23-channel coil array for high-resolution magnetic resonance imaging at 3 Tesla

**DOI:** 10.1371/journal.pone.0206963

**Published:** 2018-11-01

**Authors:** Roberta Frass-Kriegl, Lucia Isabel Navarro de Lara, Michael Pichler, Jürgen Sieg, Ewald Moser, Christian Windischberger, Elmar Laistler

**Affiliations:** Division MR Physics, Center for Medical Physics and Biomedical Engineering, Medical University of Vienna, Vienna, Austria; NYU Langone Health, UNITED STATES

## Abstract

**Purpose:**

The purpose of this work is the design, implementation and evaluation of a mechanically flexible receive-only coil array for magnetic resonance imaging (MRI) at 3 T that can be applied to various target organs and provides high parallel imaging performance.

**Methods:**

A 23-channel array was designed based on a rigid-flex printed circuit board (PCB). The flexible multi-layer part contains the copper traces forming the coil elements. The rigid part of the PCB houses the solder joints and lumped elements. The coil housing consists of rigid caps mounted above the rigid parts. Adhesive PTFE sheets cover all flexible parts. The developed array was tested on the bench as well as in phantom and in vivo MRI experiments employing parallel imaging acceleration factors up to six.

**Results:**

Efficient mutual decoupling between receive elements and detuning between receive array and body coil was achieved. An increased signal-to-noise ratio in comparison to commercial reference coils is demonstrated, especially in regions close to the developed array and for high parallel imaging acceleration factors. Exemplary in vivo images of head, ankle, knee, shoulder and hand are presented.

**Conclusion:**

Based on high sensitivity close to the array and low g-factors, this flexible coil is well suited for studies of occipital and temporal cortex, as well as musculoskeletal targets like knee, ankle, elbow and wrist.

## Introduction

Recently, the interest in mechanically flexible RF coil arrays [1–8] has grown rapidly in the MR community. The main benefit expected in comparison to rigid coils is a higher achievable signal-to-noise ratio (SNR) in case of strong variations of the targeted anatomy across patients. On the one hand, the SNR increase originates from a higher filling factor and a higher achievable ratio of unloaded to loaded quality factor when the coil is closely form-fitted to the sample. On the other hand, a flexible array is characterized by reduced inter-patient variation of Q-factor, matching, and decoupling performance because loading of each individual element is kept approximately constant by form-fitting the array to the sample. In addition, when the diameter of the individual elements is small compared to the bending diameter, element tuning is barely influenced by the bending. The combination of these properties ensures a robustly high coil performance even for large variations in patient size. Further, patient comfort can also be increased by using mechanically flexible RF coils.

Different technological approaches for the construction of flexible RF coils have been presented. For instance, geometrically adjustable transceiver arrays have been realized by making the distance between rigid coil elements variable, and introducing a “flexible” adjustable patch capacitor network between neighboring elements to change the decoupling capacitance accordingly [1]. An alternative approach has been presented by utilizing primary and secondary harmonic elements of rigid microstrip transmission lines covered by flexible fabric, where the number of elements and inter-element spacing in the array can be adjusted for each target organ [4]. Another flexible transceiver array has been proposed based on monolithic transmission line resonators decoupled by overlapping annexes fabricated on the same flexible substrate as the coil elements [6]. A flexible 64-channel receive-only array composed of rigid columns of elements attached to neighboring columns via cloth hinges has been presented, which allow neighboring columns to tilt relative to one another [3]. Alternatively, receive-only arrays with flexible individual elements have been proposed, either based on screen printed silver ink [7], or copper traces deposited on thin flexible FR4 substrate [2]. While the elements of the first array have integrated screen-printed capacitors and are mutually decoupled by coil overlap, the latter array relies on π-matching networks resulting in a double-hump resonance which is robust against variations in loading and decoupling. Further, a receive array with stretchable coil elements has been realized by sewing copper braid to stretchable fabric made of cotton and polyamide [5].

In comparison to rigid RF coils, several particular challenges come into play for the development of flexible coil arrays. Especially, the construction of suitable coil housings is more challenging for flexible coils. Housings have to provide electrical and thermal insulation for patient safety and protect coil electronics from mechanical damage, while being mechanically adjustable. Also, the materials used for the construction of the flexible coil might compromise the achievable SNR, e.g. flexible substrates with low quality factor, or increased losses due to thin conductors. The number of suitable materials is further limited by the fact that many flexible plastics are prone to be visible in MR images. In addition, high stability of the coil electronics, especially the required solder joints (e.g. for connection of coaxial cables), is required in order to achieve a robust performance even if the coil is repeatedly adjusted mechanically.

This work aims at developing a mechanically adjustable RF coil array for 3 T MRI which overcomes the prototype stage by a flexible but robust coil design, and a housing designed to allow for straightforward application in volunteer and patient studies. The array should also be suited for various target organs including the brain, as well as musculoskeletal applications, where detection sensitivity and patient comfort can be easily increased by adjusting the array to the individual anatomy. In addition, a sufficiently large number of channels in two directions should provide improved parallel imaging performance.

## Materials and methods

### Coil design and construction

The developed array is designed as a multi-layer rigid-flex printed circuit board (PCB), which consists of flexible parts containing only the copper traces of the coil elements, and rigid parts containing all required lumped element components. This way, it is possible to form-fit the array individually to each patient, while, at the same time, preventing damage of the required solder joints potentially caused by bending [9,10].

The array consists of 23 loop elements with a diameter of 45 mm each. The number and size of the coil elements were chosen according to the requirements for fMRI studies in the occipital lobe, which is one of the main target applications for the developed array. The overall size of the array was determined by the surface area needed to cover the posterior part of the head without interfering with the ears, so as to preserve patient comfort and the easy application of ear protection. The size of the individual elements was chosen in a way to provide high SNR for a penetration depth of 4 to 5 cm [11,12], and in order to operate in the sample noise dominated regime [13]. These considerations together with the choice of a hexagonal placing scheme, which allows for overlap decoupling, resulted in a total number of 23 elements arranged in five rows.

**Fig 1:**
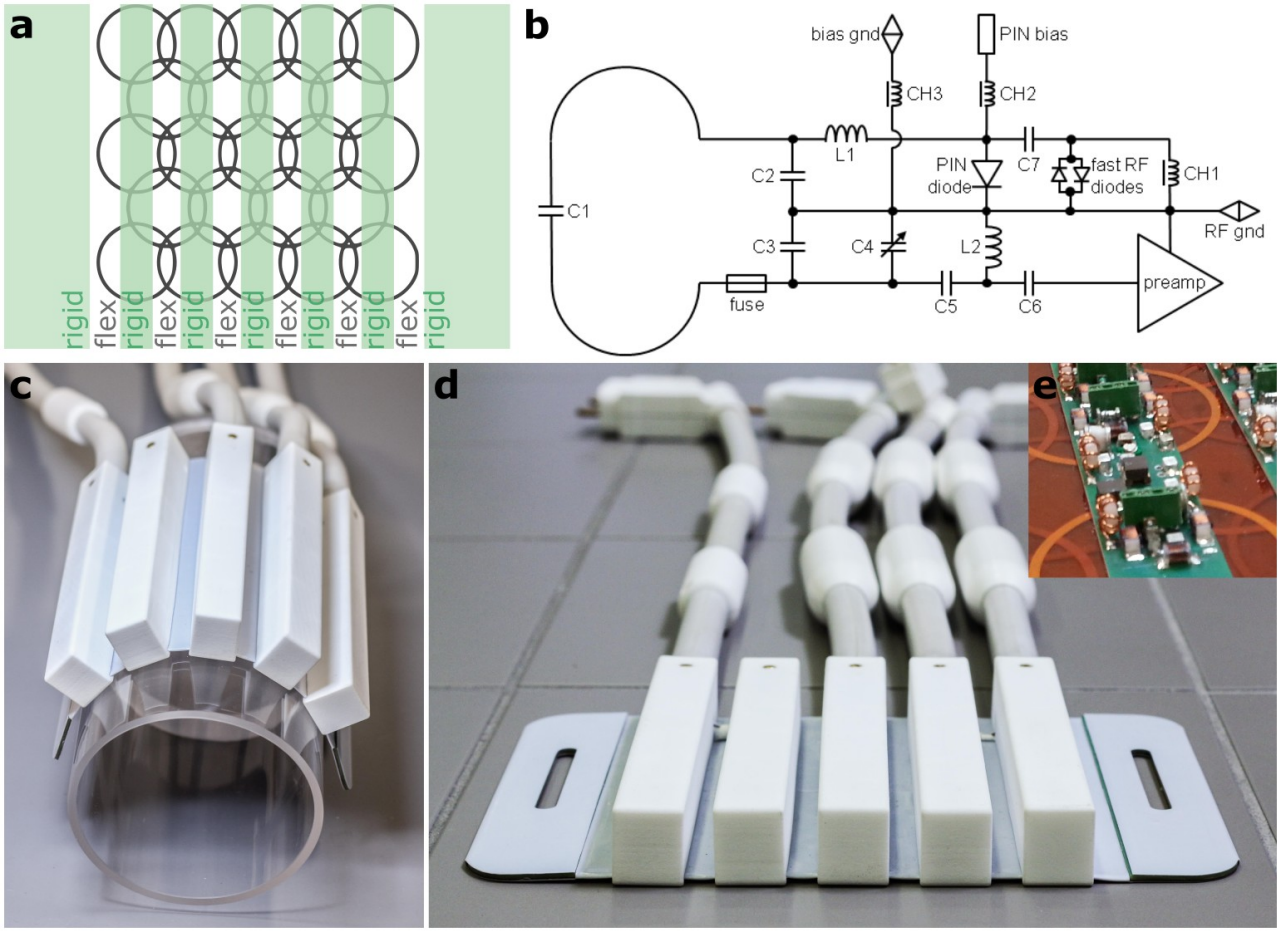
Design of the flexible 23-channel coil array. a) Schematic of the rigid-flex PCB forming the array. b) Circuit diagram for a single coil element. Photographs of the custom-built array in bent (c) and flat (d) configuration. e) Photograph showing a part of the PCB assembled with lumped element components, but without preamplifiers and cables.

The surface loops are formed by 2 mm wide copper traces deposited on thin, flexible polyimide films in three layers to enable coil overlap for mutual decoupling. On top of these flexible PCB layers rigid FR4 strips are placed (Fig 1a). On these rigid PCB parts, all lumped elements (e.g. tuning and matching capacitors) are soldered. On both lateral sides of the array, 5 cm wide rigid FR4 strips are placed for mechanical stability and for attaching fixation straps.

Adjacent coil elements are decoupled by geometrical overlap, where the distance between coil centers was set to 0.78 times the coil diameter in the PCB layout, which corresponds to the theoretically optimal overlap [14]. An experimental optimization of coil overlap was not performed. Instead, mutual decoupling is further improved employing preamplifier decoupling. For this purpose, second stage matching networks are used to connect the coil elements to low noise preamplifiers (27 ± 0.1 dB gain, 0.5 dB noise figure; Hi-Q.A. Inc., Carleton Place, Ontario, Canada) in a way that the input impedance of the preamplifier is transformed so as to minimize current flow in the coils [15]. Fig 1b shows the circuit diagram for a single coil element. The second stage matching networks are composed of the adjustable capacitor C_4_ (Murata Manufacturing Company, Kyoto, Japan), chip capacitors C_3_, C_5_ and C_6_, as well as inductor L_2_. Preamplifiers are mounted on the PCB, directly adjacent to each coil element to minimize losses between the coil and the first stage of amplification. Preamplifiers are biased with 10 V DC via the system cables connecting the coil to the MR scanner.

All elements of the receive-only array are passively and actively detunable for decoupling from the MR scanner’s body coil used for signal transmission. The detuning circuit (Fig 1b) is formed by inductor L_1_ and one of the coil’s tuning capacitors C_2_. It can be actively switched via a PIN diode (Temex Ceramics, Pessac France) biased through an RF choke CH_2_ (Coilcraft, Cumbermauld, UK). Alternatively, a passive switching mechanism was implemented consisting of fast RF diodes (Microsemi, Aliso Viejo, CA, USA), an RF choke inductor CH_1_ to short the switching currents, minimizing noise created by the fast diodes, and a 1 nF capacitor C_7_ to block direct current (DC). In addition to the detuning circuits, a series fuse with 315 mA nominal current rating is integrated in each element as safety fallback measure in case the detuning circuits should fail.

The coil housing consists of five rigid 3D-printed biocompatible polyamide caps (Fine Polyamide PA 2200, EOS GmbH—Electro Optical Systems, Krailing, Germany) placed above each rigid strip of the array for protection of the coil electronics. Adhesive PTFE sheets cover the flexible parts of the array providing a biocompatible, non-flammable, and easy-to-clean interface to the patient while maintaining mechanical flexibility (Fig 1c and 1d). The array is connected to the MR scanner via four cables of approximately 65 cm length which allows for flexible positioning of the coil on various target anatomies. Each cable is equipped with two shield current traps distributed along the cable in a way that the untrapped cable length is below one eighth of the wavelength of operation. The floating shield current trap design [16] was used, where the traps consist of split copper plated dielectric cylinders tuned with capacitors.

The total weight of the coil including all cables is 2.3 kg, of which 1.0 kg on the patient, when the cables are resting on the patient table. In comparison, the standard Siemens 4-channel flex coils (Siemens Healthcare, Erlangen, Germany) weigh 1.2/1.3 kg, of which 0.6/0.7 kg on the patient for the “Small” and the “Large” variant, respectively.

### Bench measurements

Prior to constructing and testing the complete 23-channel array, test measurements with a three-element array, i.e. the base cell of the complete array, at different bending radii were performed to evaluate the robustness of the tuning, matching, and decoupling performance against bending. Full S-parameter matrices for the test array form-fitted to one flat and three cylindrical saline phantoms (5 g/L NaCl; diameters: 105 mm, 90 mm, and 60 mm, respectively) were measured. Although 90 mm would be the minimum bending diameter for the 23-channel array preventing self-overlap, the 60 mm phantom was used to test the performance for extreme curvatures.

Upon completion of these initial tests, the 23-channel array was built and tested on the bench evaluating S-parameters, Q-factors, preamplifier decoupling and active detuning performance. Bench measurements were carried out using a two-port vector network analyzer (E5071C, Agilent, Santa Clara, USA). A spherical phantom (Siemens Healthcare, Erlangen, Germany) with an outer diameter of 180 mm and a wall thickness of 7.5 mm was used as load. The double-loop probe method [17] with a baseline value < -80 dB for the transmission S-Parameter S_21_ was used to determine the quality factors in unloaded and loaded configuration. Preamplifier decoupling and active detuning were also tested using the double-loop probe method. DC signals required for voltage supply of the preamplifiers and switching of the active detuning circuit were transmitted directly via the coil plugs connected to a custom-built coil test rig to mimic conditions in the MR scanner. The efficiency of preamplifier decoupling was quantified by comparing the S_21_ value at the Larmor frequency when the investigated coil element was either connected to the preamplifier (1.4 + 20j Ω input impedance) or terminated by a 50 Ω load (preamplifier’s noise match impedance). Active detuning was tested by holding the double probe on the same position, but actively switching the forward bias of the PIN diode on and off, and measuring the difference in S_21_ at the Larmor frequency between tuned and detuned state. The active detuning circuit was considered efficient if the produced isolation between these two states was > 35 dB.

### MRI experiments

#### MR scanner, subjects, and phantoms

MR measurements of the spherical phantom were performed on a 3 T Tim Trio whole-body scanner, which was upgraded to a Prisma Fit (Siemens Healthcare, Erlangen, Germany) before in vivo experiments and measurements with the cylindrical phantom. Eight healthy subjects (all male, age: 34.5 ± 6.6 years) participated in in vivo experiments after giving written informed consent. The study was approved by the local ethics board and conducted according to the Declaration of Helsinki. The study has been approved by the Ethics Committee of the Medical University of Vienna, EK2014/2016.

#### Phantom experiments

MR experiments with the spherical phantom were used to verify the compliance of the developed coil with the safety requirements outlined in the IEC guideline 60601-2-33. One patient risk to be addressed is tissue heating due to electrical fields produced during RF transmission, i.e. specific absorption rate (SAR). Since the developed coil is a receive-only array, it must be ensured that it does not cause distortions, in particular local concentration, of the electromagnetic field generated by the MR scanner’s body transmit coil. This kind of distortions could occur in case of insufficient isolation between receive-only and transmit coil during RF transmission. Therefore, the isolation between the developed array and the MR scanner’s body transmit coil was investigated by acquiring 3D gradient echo images and B_1_^+^ maps with and without the receive-only array present using the body coil for both, transmission and reception. Consequently, the acquired images where visually inspected for artifacts potentially caused by the receive-only array and ratio maps of the measured B_1_^+^ distributions were calculated. Secondly, the patient risk due to high surface temperatures of the developed coil during scanning was investigated. For this purpose, a 20-min test sequence employing a high transmit field B_1_^+^ at the maximum SAR allowed by the scanner (as suggested in the IEC guideline 60601-2-33, Annex AA, AA.1, subclause Concerning 201.12.4.103.1 Limits of temperature) was run for two different conditions: once with the coil on top of the phantom in the isocenter, and once for an off-center position (i.e. moved towards the wall of the scanner bore and shifted by 5 cm along the z-axis). During these measurements, the surface temperature of the array on the patient side was monitored using two fiber optic probes (model T1; Neoptix Inc., Quebec, Canada); temperature sensors were placed directly below locations of the detuning circuits on the PCB, one at the center, and the other at the side of the array.

As fMRI studies of the occipital lobe are one of the main target applications for the developed array, the achievable performance was compared to a commercially available head coil. The posterior part (consisting of 20 channels) of the widely used 32-channel head coil (Siemens Healthcare, Erlangen, Germany) was used as reference. The reference coil is a split-type version (12 anterior and 20 posterior coil elements) of the 32-channel array set in the soccer-ball geometry as described by Wiggins et al. [18]. The performance of the arrays loaded by the spherical phantom in terms of SNR and GRAPPA g-factor was evaluated by applying the pseudo multiple replica method [19] and off-line GRAPPA reconstruction as described by Breuer et al. [20]. Therefore, noise-only data, for computing the noise correlation matrix, and fully encoded transversal 2D gradient echo images (T_R_/T_E_ = 500 ms/3.34 ms, 25° flip angle, 192 x 192 acquisition matrix, 1 x 1 mm^2^ in-plane resolution, 60 slices, 3 mm slice thickness) were acquired. Acceleration factors of R = 1 (no acceleration), R = 2, R = 3, and R = 4 were mimicked during reconstruction by eliminating respective phase encoding steps. SNR maps were calculated for all investigated acceleration factors, and SNR ratios were used to extract g-factor maps. In vivo brain images (T_R_/T_E_ = 500 ms/3.34 ms, 25° flip angle, 192 x 192 acquisition matrix, 1 x 1 mm^2^ in-plane resolution, 60 slices, 3 mm slice thickness) were acquired with both coils, and corresponding SNR maps were calculated again using the pseudo multiple replica method.

To investigate the applicability of the developed flexible array for musculoskeletal imaging targets (e.g. knee), MR measurements with the array form-fitted to a cylindrical phantom (diameter 12 cm, length 20 cm) were performed. As described above, SNR- and g-factor maps were calculated based on gradient echo images (T_R_/T_E_ = 470 ms/3.23 ms, 20° flip angle, transversal: 192 x 192 acquisition matrix, sagittal: 192 x 288 acquisition matrix, 1 x 1 mm^2^ in-plane resolution, 3 mm slice thickness, 510 Hz/Px), and noise-only acquisitions. Acceleration factors of R = 1, 2, 3, 4, 6, and 8 were investigated. The performance of the novel array was compared to the “Small” version of the standard Siemens 4-channel flex coil (Siemens Healthcare, Erlangen, Germany).

#### In vivo experiments

To demonstrate its versatility in vivo, the developed array was used to acquire high-resolution magnetization prepared rapid gradient-echo (MPRAGE) images of the occipital brain (2 volunteers), as well as turbo spin echo (TSE) images of the knee (3 volunteers), the hand and wrist (1 volunteer), the shoulder (1 volunteer), and the ankle (2 volunteers) of volunteers (note that one volunteer participated in two sessions). Scans of the head, knee, ankle and shoulder were performed with the volunteers in supine position, while images of the hand and wrist were acquired in prone position. For brain imaging, subjects were lying on the developed array which was bent towards the temples. For ankle imaging, the volunteers’ heel was resting on the array which was then wrapped around the ankle. For imaging of the hand and wrist the array was positioned on the dorsal side of the volunteer’s hand which was resting on the patient table. For knee imaging the array was laterally wrapped around the volunteers’ knee, without fully surrounding it. For imaging of the shoulder the array was positioned anterior of the volunteer’s shoulder and form fitted to the side of the arm in a way than the volunteer did not lie on the coil. Sequence parameters are summarized in Table 1.

**Table 1:** Imaging parameters for the in vivo acquisitions shown in Fig 7.

organ	a) brain	b) ankle	c) hand	d) knee			e) shoulder	
plane	coronal	sagittal	coronal	axial	coronal	sagittal	axial	coronal
**sequence**	MP RAGE	TSE	TSE	TSE	TSE	TSE	TSE	TSE
**T**_**R**_ **[ms]**	2000	3150	1160	7010	7010	4000	4510	4780
**T**_**E**_ **[ms]**	2.9	15	16	32	32	85	17	17
**T**_**I**_ **[ms] or turbo factor**	TI 933	TF 7	TF 5	TF 6	TF 6	TF 11	TF 6	TF 6
**BW / pix. [Hz]**	200	145	203	195	195	205	195	195
**R**	2	2	3	2	2	2	2	2
**in-plane res. [μm]**	170 x 170	260 x 260	200 x 200	320 x 250	320 x 250	420 x 340	180 x 180	260 x 260
**matrix**	648 x 768	486 x 576	672 x 1024	410 x 512	410 x 512	307 x 384	864 x 1024	528 x 704
**slice th. [mm]**	1.75	1.5	1.5	2.5	2.5	3	1.5	1.5
**slices**	88	37	16	33	30	25	35	37
**averages**	1	1	2	1	1	1	1	1
**T**_**acq**_ **[min]**	06:17	02:15	03:25	05:08	05:08	01:52	04:44	06:03

To demonstrate the applicability of higher parallel imaging acceleration factors in vivo with the developed array, SNR- and g-factor maps were calculated based on gradient echo images (T_R_/T_E_ = 470 ms/3.23 ms, 20° flip angle, brain: 288 x 288 acquisition matrix, knee: 192 x 288 acquisition matrix, 1 x 1 mm^2^ in-plane resolution, 3 mm slice thickness, 510 Hz/Px), and noise-only acquisitions as described above for brain and knee data. Acceleration factors of R = 1, 2, 3, 4, and 6 were investigated.

## Results

### Bench measurements

Initial tests with the three-element array showed that the tuning, matching and decoupling performance of the array is robust against variations in the bending diameter. When the test array was either loaded by the 90 mm or 105 mm cylindrical phantom, or the flat phantom, decoupling was better than -9.9 dB (without preamplifier decoupling) and matching better than -11.4 dB. For the 60 mm cylindrical phantom representing a curvature out of specification for the developed array, coupling increased to -8 dB, whereas matching was below -14.5 dB. S-parameters for all configurations are given in Table 2. The resonance frequency of the three array elements varied by ± 0.2 MHz around the target Larmor frequency of 123.2 MHz for the bending radii investigated.

**Table 2:** S-parameters of a three-element test array as function of the bending diameter.

Bending diameter	S_11_ [dB]	S_22_ [dB]	S_33_ [dB]	S_12_ [dB]	S_13_ [dB]	S_23_ [dB]
**Flat**	-17.3	-11.4	-13.6	-16.9	-10.5	-19.6
**105 mm**	-17.3	-27.7	-16.7	-14.0	-10.9	-15.8
**90 mm**	-18.1	-23.2	-25.5	-14.0	-9.9	-16.1
**60 mm**	-31.5	-27.3	-14.5	-8.1	-8.0	-13.0

**Fig 2:**
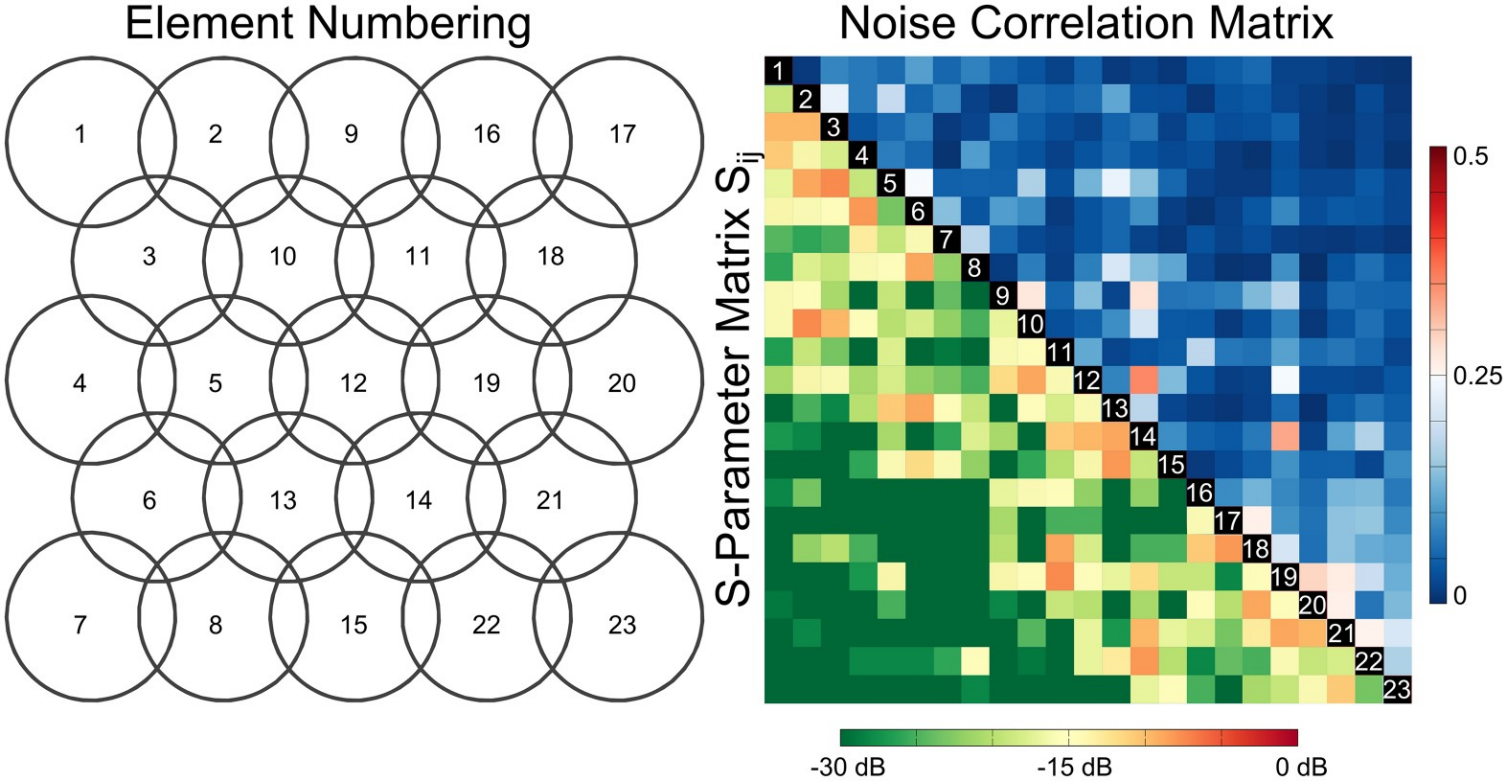
S-parameter and noise correlation matrix of the 23-channel array. The arrangement and numbering of the individual array elements are shown on the left. The S-parameter matrix measured on the bench without preamplifier decoupling and the normalized noise correlation matrix are shown on the right. Matching better than -16 dB and decoupling better than -8 dB was achieved for all elements. Mean and maximum off-diagonal noise correlation values are 6.6% and 35%, respectively.

All coil elements of the 23-channel array form-fitted to the spherical phantom were tuned to the Larmor frequency of interest, and matching levels below -16 dB were achieved for all elements (Fig 2). Mutual decoupling via overlap decoupling alone was below -8 dB; an additional isolation of at least -16 dB was achieved by preamplifier decoupling. Switching the active detuning network generated more than 40 dB isolation for all elements. The blocking efficiency of the cable traps was better than -14 dB. For an isolated single coil element the measured Q-factor was 152 when unloaded, and 67 when loaded, respectively. In array configuration, the unloaded and loaded Q-factors dropped to 130, and 60, respectively.

### MRI experiments

For experiments with the developed array and the spherical phantom the mean measured noise correlation was 6.6 ± 6.0%, with a maximum value of 35% (Fig 2). The receive-only array is well isolated from the transmitting MR body coil, which ensures patient safety and avoids transmit field distortion. The maximum relative deviation in B_1_^+^ maps acquired with the body coil with and without the receive array present was below 10%. During the 20-min maximum SAR test sequence, the surface temperature of the developed array increased from 21.0°C to 23.4°C for the isocenter position, and to 24.2°C for the off-center position, respectively. Both values are well below the maximum allowable temperature rise of 4°C stated in IEC 60601-2-33, Annex AA, AA.1, subclause Concerning 201.12.4.103.1 Limits of temperature, and the maximum temperature of 43 °C for skin contact specified in 60601–1, subclause 11.1.2, table 24.

**Fig 3:**
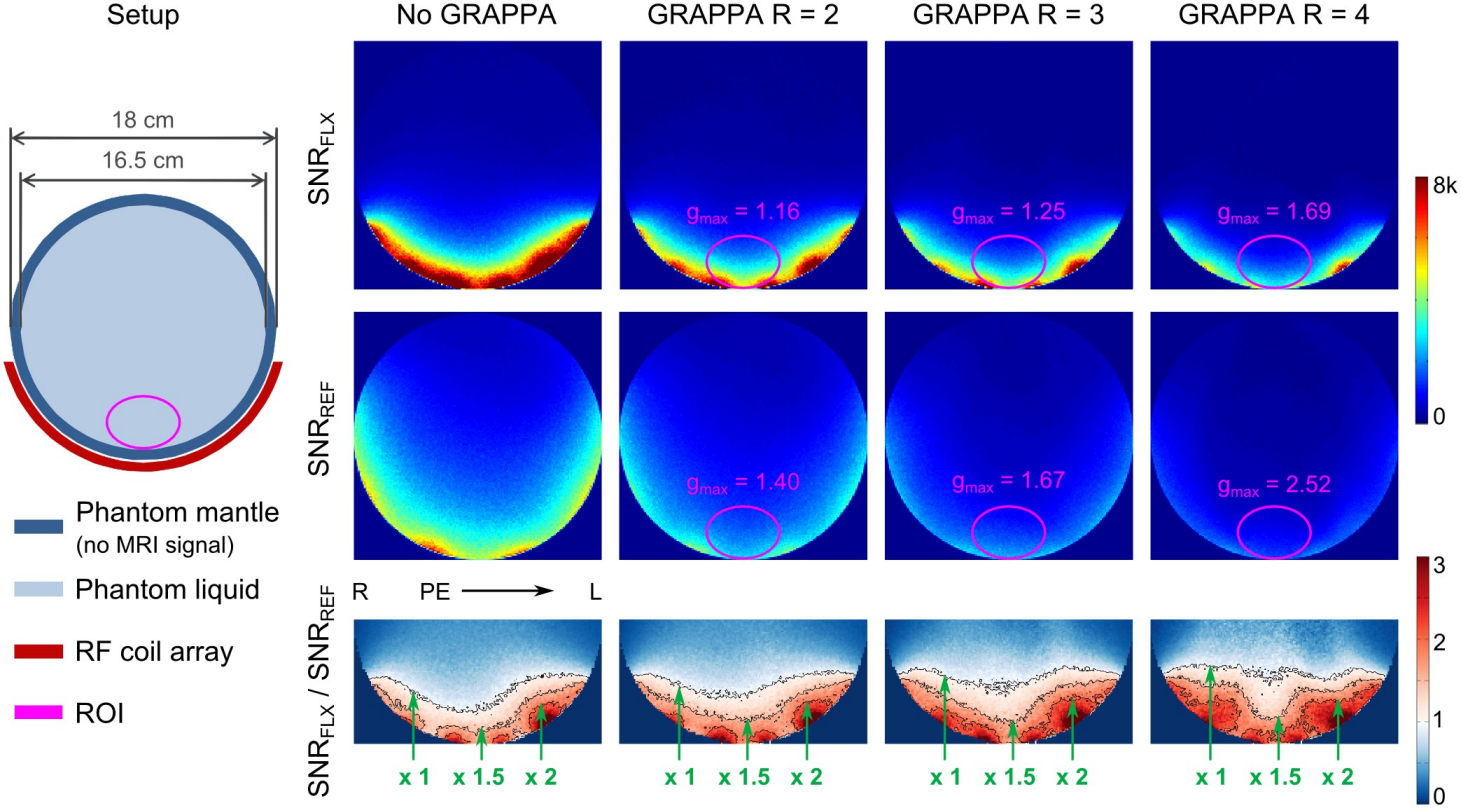
Setup for phantom MRI experiments and SNR data. Left: The setup for MRI experiments with the array and a spherical phantom. Right: Comparison between the developed array (FLX) and the reference coil (REF) in terms of SNR with GRAPPA acceleration factors R = 1 (no acceleration), 2, 3, and 4. Maximum g-factors in an elliptical ROI representing the occipital lope are given, and corresponding SNR-ratio maps are shown in the bottom row.

**Fig 4:**
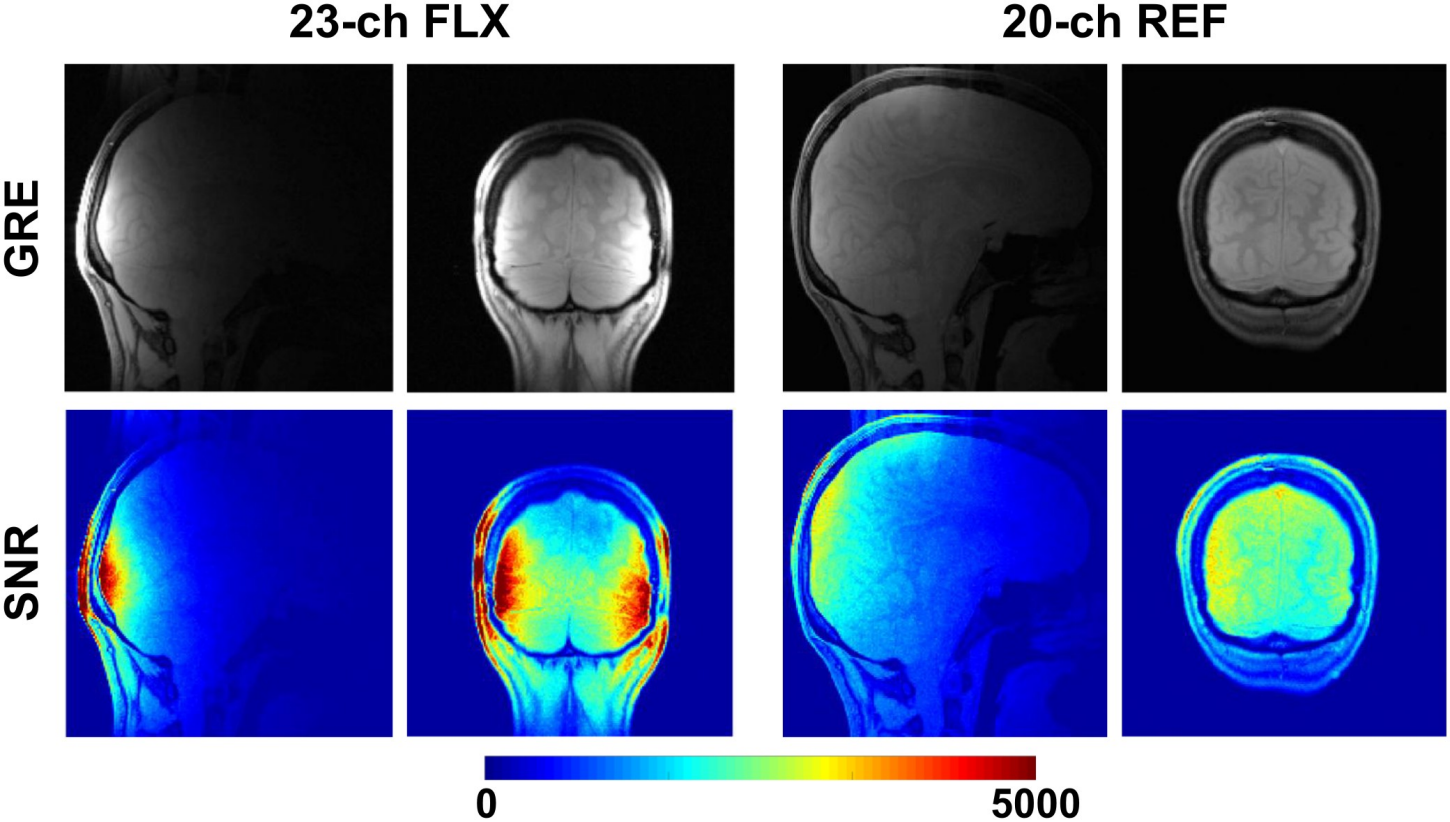
Brain images acquired with the developed 23-channel array in comparison to a 20-channel reference coil. The same image windowing and color scales were used for both compared arrays.

The comparison of the developed array to the reference head coil is presented in Figs 3 and 4. SNR maps (Fig 3) computed for GRAPPA acceleration factors of R = 1–4 show a significant SNR gain in comparison to the reference coil in the region close to the coil especially for high acceleration factors. The maximum g-factor in an elliptical ROI representing the occipital lobe increased from 1.16 to 1.69 for R increasing from 2 to 4. In contrast, the maximum g-factor in the same ROI increased from 1.40 to 2.52 for the reference coil. Fig 4 shows in vivo brain images (GRE) and corresponding SNR maps for the two arrays, demonstrating the high SNR of the developed 23-channel array in the occipital lobe.

**Fig 5:**
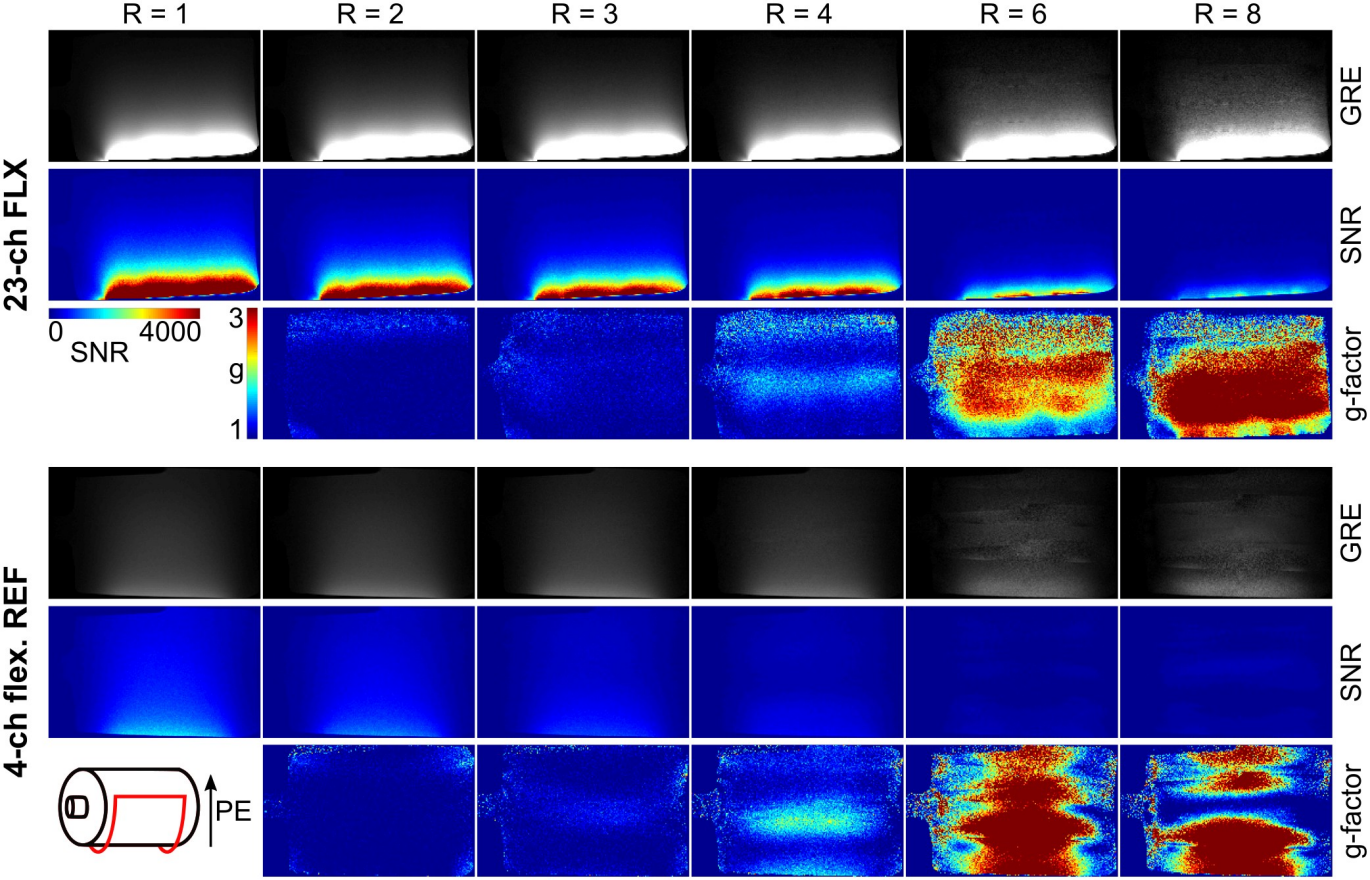
Saggital gradient echo (GRE) images with corresponding SNR- and g-factor maps comparing the developed 23-channel array to a 4-channel flexible reference coil. R is the applied GRAPPA acceleration factor; PE is the phase encoding direction. The same image windowing and color scales were used for both compared arrays. The sketch in the lower left corner shows the positioning of the coil on the phantom.

**Fig 6:**
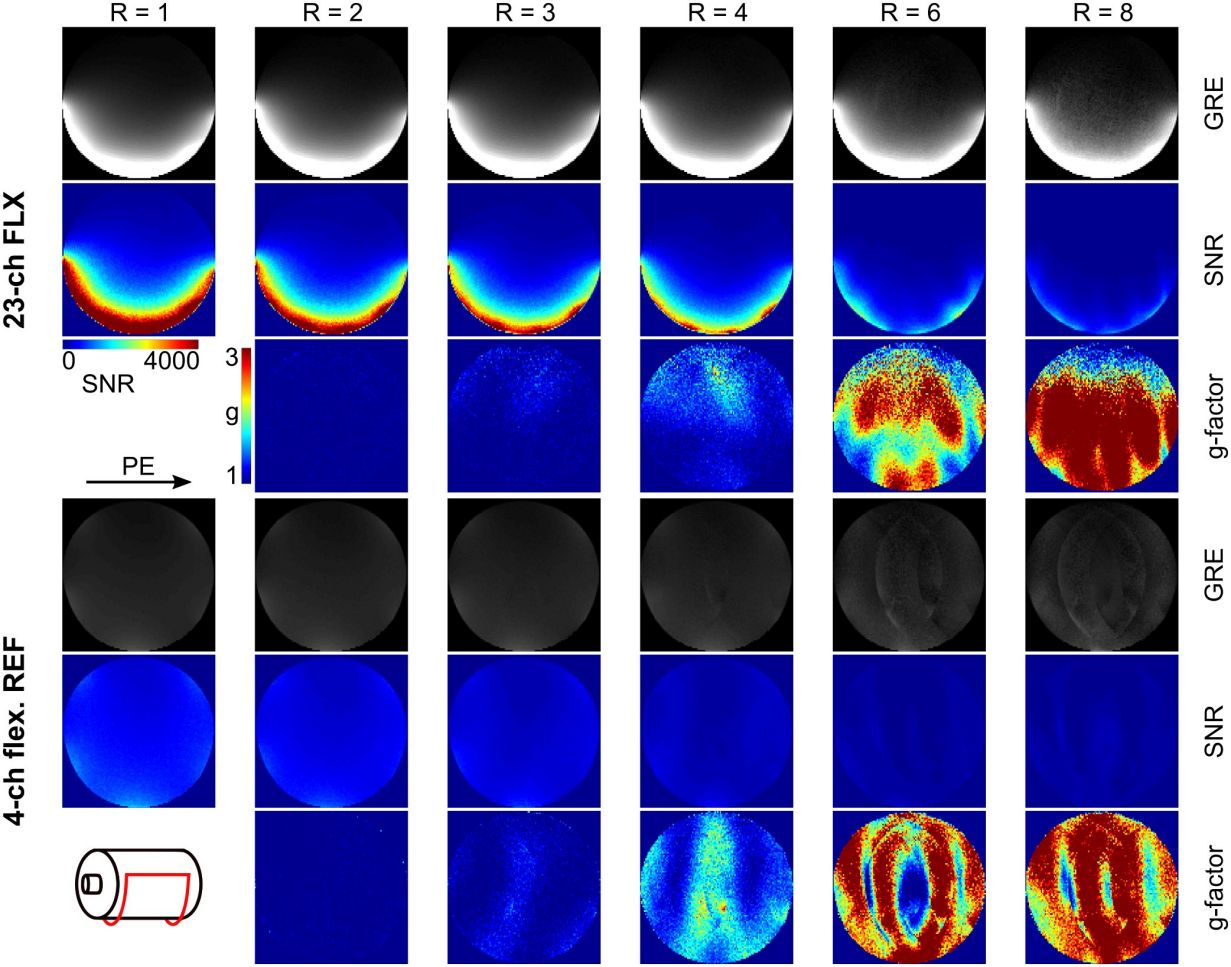
Transversal gradient echo (GRE) images with corresponding SNR- and g-factor maps comparing the developed 23-channel array to a 4-channel flexible reference coil. R is the applied GRAPPA acceleration factor; PE is the phase encoding direction. The same image windowing and color scales were used for both compared arrays. The sketch in the lower left corner shows the positioning of the coil on the phantom.

The comparison of the developed flex-array to the flexible reference coil is presented in Fig 5 (sagittal view) and Fig 6 (transversal view). With the developed array, clearly higher SNR and lower g-factors are achieved for all investigated acceleration factors (R = 1, 2, 3, 4, 6, 8). Parallel imaging acceleration factor up to 4 resulted in artifact-free reconstructed images. For R = 6, accurate image reconstruction is achieved in regions with high SNR, i.e. target regions close to the array, while more distant regions start to show reconstruction artifacts. Results for R = 8 demonstrate the point of failure for GRAPPA reconstructions with the developed array, as expected for the chosen layout (see Fig 2) with maximum five coil elements along one direction.

**Fig 7:**
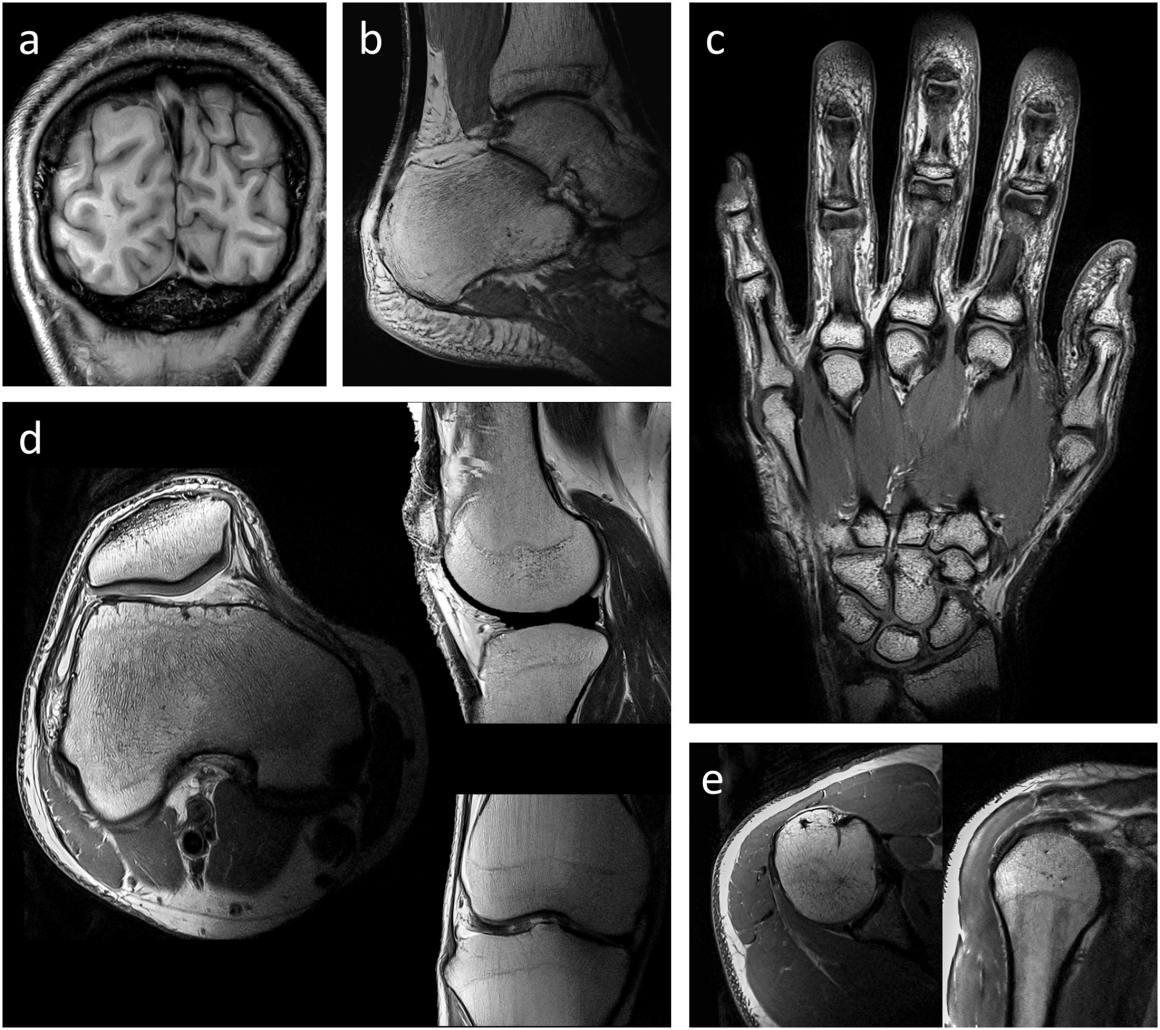
In vivo MR images. MR images showing image quality and versatility of the flexible coil array. a) occipital cortex, b) ankle, c) hand, d) knee in axial (left), sagittal (top right), and coronal (bottom right) view, e) shoulder in axial (left) and coronal (right) view. The corresponding imaging parameters are listed in Table 1.

The coil array was successfully form-fitted to all body parts investigated. In vivo images with very high spatial resolution (i.e. 170 to 420 μm in-plane resolution), employing GRAPPA factors of up to 3, were acquired in measurement times between 2 and 6 minutes per scan, and are shown in Fig 7. The acquired images demonstrate the versatility of the developed array for investigating various anatomical targets with high SNR and adequate coverage.

**Fig 8:**
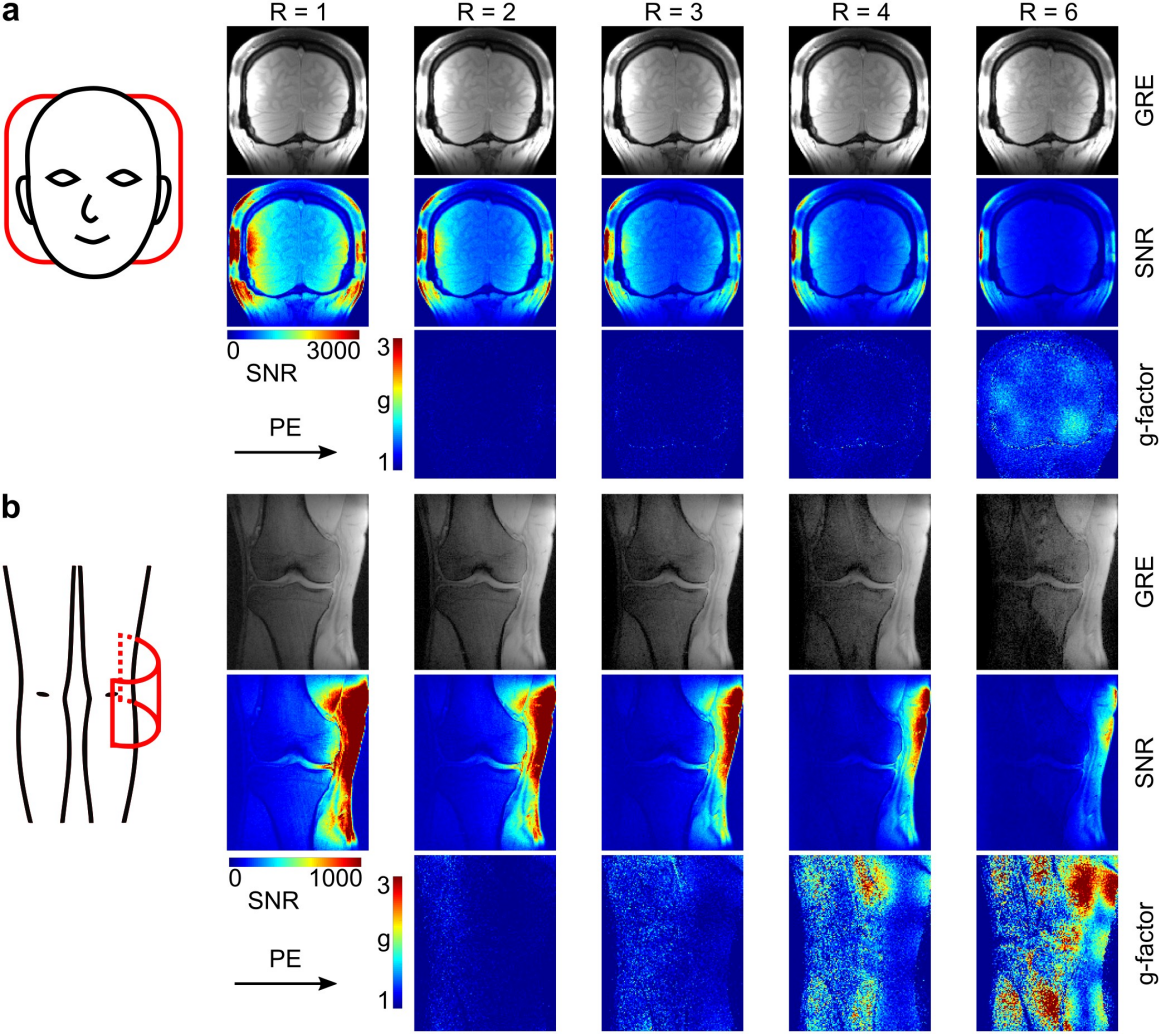
In vivo gradient echo (GRE) images with corresponding SNR- and g-factor maps of brain (a) and knee (b). R is the applied GRAPPA acceleration factor; PE is the phase encoding direction. The sketches on the left corner show the positioning of the coil on the subject. As indicated in the sketch, the array does not fully surround the volunteer’s knee, which is the reason for the signal drop-off in the medial region.

Fig 8 shows images, SNR- and g-factor maps for in vivo brain and knee data acquired with the developed array. For coronal brain images with phase encoding form right to left, acceleration factors up to R = 6 result in artifact free reconstructed images, and g-factors below 2. In contrast, data from coronal knee acquisitions show reconstruction artifacts and a strong SNR decrease due to high g-factors (> 3) already for acceleration factors of R = 4 and R = 6. This can be explained by the positioning of the array on the subject and the choice of the phase encoding direction, as shown in the sketches on the left in Fig 8.

## Discussion and conclusion

In this work, a novel, mechanically flexible RF coil array for MRI at 3 T has been developed and tested in phantom and in vivo experiments. The design of the array as a rigid-flex printed circuit board (PCB) enables adjusting the array to various anatomical sites individually for each patient, while, at the same time, preventing damage of the required solder joints potentially caused by bending. It was demonstrated that the array can be form-fitted to various target anatomies with different bending radii, thereby maintaining high SNR and parallel imaging performance. This robustness of the coil performance could be expected due to the small diameter of the individual coil elements in comparison to the used bending radii.

The developed coil array features bending along one coil dimension, which enables optimal form-fitting for target anatomies that can be roughly approximated by cylindrical geometries, e.g. knee or elbow. The rigid PCB strips and the rigid housing caps provide mechanical stability and protection for the array and, therefore, increase user-friendliness and ease-of-use; however, they also prevent bending along the second coil dimension. Moderate two-dimensional flexibility would become possible by intersecting the rigid PCB strips and providing a separate housing for each strip part, with cable connections between them. However, for the coil presented in this work, the rigid PCB strips are densely populated with components required for the functionality of the coil, which makes a further reduction of the rigid PCB area impossible. Nevertheless, this strategy could be pursued for coil arrays with larger individual elements. Further, a coil array that is truly adjustable in two dimensions would require different coil technology so as to enable stretching as well as bending deformations of the coil elements, in order to prevent buckle formation. Although such technology was already presented [5,21], the design of coil housings which are robust and fulfill all safety requirements becomes even more challenging.

Measurements of the Q-factor indicate that the coil size chosen in this work approaches the minimum coil size for which sample noise dominates over coil noise at the given Larmor frequency. The small element size was chosen as the presented coil array was primarily designed for imaging targets at 4 to 5 cm depth. The observed Q-drop due to the presence of neighboring coil elements can be explained by residual inter-element coupling and eddy current losses in the conductors of the neighboring elements [11]. While these losses are expected to be reduced for wire coils, flat strip conductors were chosen because of their compatibility with the presented PCB approach.

The long-term durability of the employed materials for PCB and coil housing has yet to be investigated. The rigid-flex PCB alone is specified for > 10000 bending cycles by the manufacturer in case of bending radii larger than 250 times the thickness of the flexible substrate (i.e. 75 mm for the presented array), which is typically fulfilled for MR applications. An alternative housing material, in case the adhesion of the PTFE sheets should deteriorate too quickly, could be vinyl fabric [22], which can be sewed instead of glued.

The coil’s high sensitivity as well as its high potential for parallel imaging applications is demonstrated in comparison to a commercial head coil with a similar number of channels and a commercial flexible array with a lower number of elements. The reference coils provide better signal homogeneity but are clearly outperformed by the developed array in terms of SNR and g-factors for a penetration depth of up to 5 cm within the sample.

Based on the very high SNR close to the coil, the developed array is well suited for studies of the occipital cortex. The presented coil array has recently been shown to allow for significant performance gain in high resolution diffusion weighted imaging [23] and multi-parametric mapping of the occipital cortex [24]. Other potential applications include retinotopic and population receptive field (pRF) mapping [25–27]. A key advantage of the present coil is the excellent performance in parallel imaging applications, especially for high acceleration or multiband factors [28,29]. Further, the developed coil can be conveniently used in combination with other devices for auditory or visual stimulation as it can be form-fitted to the back of the head in a way that is comfortable for the patient, without interfering with the ears or the visual field.

Alternatively, the developed array is also well suited for musculoskeletal applications, which typically exhibit a large number of small, clinically relevant anatomical structures and, thus, require high SNR. The versatility of the device in this respect has been demonstrated by acquiring high-resolution images of various anatomical targets employing parallel imaging methods. As human extremities vary strongly in size and shape, these applications will benefit particularly from the form-fitting capability of the array. Additionally, pediatric MR examinations, e.g. [30], present a promising field of application for the developed coil array, because size variations are even larger than in adults, and short scan durations are of particular importance in addition to uncomplicated handling and patient comfort.

## References

[1] Adriany G, van de Moortele P-F, Ritter J, Moeller S, Auerbach EJ, Akgün C, et al A geometrically adjustable 16-channel transmit/receive transmission line array for improved RF efficiency and parallel imaging performance at 7 Tesla. Magn Reson Med. 2008;59: 590–597. 10.1002/mrm.21488 18219635

[2] Nordmeyer-Massner JA, De Zanche N, Pruessmann KP. Mechanically adjustable coil array for wrist MRI. Magn Reson Med. 2009;61: 429–38. 10.1002/mrm.21868 19161134

[3] Hardy CJ, Giaquinto RO, Piel JE, Rohling KW, Marinelli L, Blezek DJ, et al 128-channel body MRI with a flexible high-density receiver-coil array. J Magn Reson imaging. 2008;28: 1219–25. 10.1002/jmri.21463 18972330

[4] Wu B, Zhang X, Wang C, Li Y, Pang Y, Lu J, et al Flexible transceiver array for ultrahigh field human MR imaging. Magn Reson Med. 2012;68: 1332–1338. 10.1002/mrm.24121 22246803 PMC3350759

[5] Nordmeyer-Massner JA, De Zanche N, Pruessmann KP. Stretchable coil arrays: Application to knee imaging under varying flexion angles. Magn Reson Med. 2012;67: 872–879. 10.1002/mrm.23240 22213018

[6] Kriegl R, Ginefri J-C, Poirier-Quinot M, Darrasse L, Goluch S, Kuehne A, et al Novel inductive decoupling technique for flexible transceiver arrays of monolithic transmission line resonators. Magn Reson Med. 2015;73: 1669–1681. 10.1002/mrm.25260 24753115

[7] Corea JR, Flynn AM, Lechêne B, Scott G, Reed GD, Shin PJ, et al Screen-printed flexible MRI receive coils. Nat Commun. 2016;7: 10839 10.1038/ncomms10839 26961073 PMC5553354

[8] Zhang B, Sodickson DK, Cloos MA. A high-impedance detector-array glove for magnetic resonance imaging of the hand. Nat Biomed Eng. 2018; 10.1038/s41551-018-0233-y PMC6405230 30854251

[9] Kriegl R, Navarro de Lara LI, Pichler M, Sieg J, Moser E, Windischberger C, et al. Flexible 23-Channel RF Coil Array for fMRI Studies of the Occipital Lobe at 3T. Proceedings of 21st Annual Meeting of OHBM. Honolulu, Hawaii, USA; 2015. p. 1865.

[10] Kriegl R, Navarro de Lara LI, Pichler M, Sieg J, Moser E, Windischberger C, et al. Flexible 23-Channel Receive-Only Coil Array for High-Resolution 3T MRI. Proceedings of the 33rd Annual Meeting of ESMRMB. Vienna, Austria; 2016. p. 328.

[11] Kumar A, Edelstein WA, Bottomley PA. Noise figure limits for circular loop MR coils. Magn Reson Med. 2009;61: 1201–1209. 10.1002/mrm.21948 19253376 PMC2869245

[12] Roemer PB, Edelstein WA, Hayes CE, Souza SP, Mueller OM. The NMR phased array. Magn Reson Med. 1990;16: 192–225. 2266841 10.1002/mrm.1910160203

[13] Darrasse L, Ginefri J-C. Perspectives with cryogenic RF probes in biomedical MRI. Biochimie. 2003;85: 915–937. 14652180 10.1016/j.biochi.2003.09.016

[14] Mispelter J, Lupu M, Briguet A. NMR Probeheads for Biophysical and Biomedical Experiments: Theoretical Principles and Practical Guidelines. London: Imperial College Press; 2006.

[15] Reykowski A, Wright SM, Porter JR. Design of matching networks for low noise preamplifiers. Magn Reson Med. 1995;33: 848–852. 7651124 10.1002/mrm.1910330617

[16] Seeber DA, Jevtic J, Menon A. Floating shield current suppression trap. Concepts Magn Reson Part B Magnertic Reson Eng. 2004;21B: 26–31.

[17] Darrasse L, Kassab G. Quick measurement of NMR-coil sensitivity with a dual-loop probe. Rev Sci Instrum. 1993;64: 1841–1844.

[18] Wiggins GC, Triantafyllou C, Potthast A, Reykowski A, Nittka M, Wald LL. 32-channel 3 Tesla receive-only phased-array head coil with soccer-ball element geometry. Magn Reson Med. 2006;56: 216–223. 10.1002/mrm.20925 16767762

[19] Robson PM, Grant AK, Madhuranthakam AJ, Lattanzi R, Sodickson DK, McKenzie CA. Comprehensive quantification of signal-to-noise ratio and g-factor for image-based and k-space-based parallel imaging reconstructions. Magn Reson Med. 2008;60: 895–907. 10.1002/mrm.21728 18816810 PMC2838249

[20] Breuer FA, Kannengiesser SAR, Blaimer M, Seiberlich N, Jakob PM, Griswold MA. General formulation for quantitative g-factor calculation in GRAPPA reconstructions. Magn Reson Med. 2009;62: 739–746. 10.1002/mrm.22066 19585608

[21] Gruber B, Zink S. Anatomically adaptive local coils for MRI Imaging–Evaluation of stretchable antennas at 1.5T. Proceedings of the 24th Annual Meeting of the ISMRM. 2016. p. 543.

[22] Erturk MA, Raaijmakers AJE, Adriany G, Ugurbil K, Metzger GJ. A 16-channel combined loop-dipole transceiver array for 7 Tesla body MRI. Magn Reson Med. 2017;77: 884–894. 10.1002/mrm.26153 26887533 PMC4988950

[23] Kirilina E, Attar FM, Edwards LJ, Pine K, Weiskopf N. High resolution in vivo diffusion weighted imaging of the human occipital cortex: enabled by 300mT/m gradients and flexible radio-frequency surface coils. Proceedings of 26th Annual Meeting of the ISMRM. 2018. p. 1617.

[24] Pine K, Vaculciakova L, Kirilina E, Scherf N, Weiskopf N. Multi-Parameter Mapping with 500 μm Resolution Using a Flexible 23-Channel RF Coil. Proceedings of the 26th Annual Meeting of the ISMRM. 2018. p. 2760.

[25] Dumoulin SO, Wandell BA. Population receptive field estimates in human visual cortex. Neuroimage. 2008;39: 647–660. 10.1016/j.neuroimage.2007.09.034 17977024 PMC3073038

[26] Hummer A, Ritter M, Tik M, Ledolter AA, Woletz M, Holder GE, et al Eyetracker-based gaze correction for robust mapping of population receptive fields. Neuroimage. Elsevier B.V.; 2016;142: 211–224. 10.1016/j.neuroimage.2016.07.003 27389789

[27] Boubela RN, Kalcher K, Nasel C, Moser E. Scanning fast and slow: current limitations of 3 Tesla functional MRI and future potential. Front Phys. 2014;2: 1–8. 10.3389/fphy.2014.00001 PMC5291320 28164083

[28] Larkman DJ, Hajnal J V., Herlihy AH, Coutts GA, Young IR, Ehnholm G. Use of multicoil arrays for separation of signal from multiple slices simultaneously excited. J Magn Reson Imaging. 2001;13: 313–317. 11169840 10.1002/1522-2586(200102)13:2<313::aid-jmri1045>3.0.co;2-w

[29] Moeller S, Yacoub E, Olman CA, Auerbach E, Strupp J, Harel N, et al Multiband multislice GE-EPI at 7 tesla, with 16-fold acceleration using partial parallel imaging with application to high spatial and temporal whole-brain FMRI. Magn Reson Med. 2010;63: 1144–1153. 10.1002/mrm.22361 20432285 PMC2906244

[30] Keil B, Alagappan V, Mareyam A, McNab J a, Fujimoto K, Tountcheva V, et al Size-optimized 32-channel brain arrays for 3 T pediatric imaging. Magn Reson Med. 2011;66: 1777–87. 10.1002/mrm.22961 21656548 PMC3218247

